# The Possible Causal Link of Periodontitis to Neuropsychiatric Disorders: More Than Psychosocial Mechanisms

**DOI:** 10.3390/ijms20153723

**Published:** 2019-07-30

**Authors:** Sadayuki Hashioka, Ken Inoue, Tsuyoshi Miyaoka, Maiko Hayashida, Rei Wake, Arata Oh-Nishi, Masatoshi Inagaki

**Affiliations:** 1 Department of Psychiatry, Shimane University, 89-1 Enya-cho, Izumo 693-8501, Japan; 2 Health Service Center, Kochi University, 2-5-1 Akebono-cho, Kochi 780-8520, Japan

**Keywords:** periodontitis, neuropsychiatric disorders, Alzheimer’s disease, Parkinson’s disease, major depression, schizophrenia, neuroinflammation, microglia

## Abstract

Increasing evidence implies a possible causal link between periodontitis and neuropsychiatric disorders, such as Alzheimer’s disease (AD) and major depression (MD). A possible mechanism underlying such a link can be explained by neuroinflammation induced by chronic systemic inflammation. This review article focuses on an overview of the biological and epidemiological evidence for a feasible causal link of periodontitis to neuropsychiatric disorders, including AD, MD, Parkinson’s disease, and schizophrenia, as well as the neurological event, ischemic stroke. If there is such a link, a broad spectrum of neuropsychiatric disorders associated with neuroinflammation could be preventable and modifiable by simple daily dealings for oral hygiene. However, the notion that periodontitis is a risk factor for neuropsychiatric disorders remains to be effectively substantiated.

## 1. Introduction

Periodontitis is a chronic, oral multi-bacterial infection affecting nearly 50% of the population worldwide and is the most prevalent inflammatory disease in adults [1,2]. Periodontitis is not only an oral localized inflammatory disease, but also elicits low-grade systemic inflammation via both the release of pro-inflammatory cytokines and the invasion of periodontitis bacteria (e.g., *Porphyromonas gingivalis* (*P. gingivalis*)) along with their components (e.g., lipopolysaccharide (LPS) and flagellin) into systemic circulation [3]. Periodontitis thus causes or hastens other chronic systemic inflammatory diseases, including atherosclerosis, cardiovascular diseases, diabetes, and rheumatoid arthritis [4]. In particular, the causal link between periodontitis and infective endocarditis has been known for many decades. Besides inducing systemic inflammation, increasing evidence implies that periodontitis provokes chronic inflammation associated with activation of microglia, the immune cells in the brain, which is referred to as neuroinflammation (reviewed in [5,6,7]). 

The concept of neuroinflammation was originally proposed for neurodegenerative disorders in the 1980s, based on two historical discoveries. The first was the immunohistochemical identification of activated microglia in association with the lesions in Alzheimer’s disease (AD) brains [8]. The second was the epidemiologic finding that rheumatoid arthritics, who regularly consume anti-inflammatory agents, were relatively spared from AD [9]. In the ensuing years, activated microglia have also been found in the lesions of Parkinson’s disease (PD), multiple sclerosis, and amyotrophic lateral sclerosis [10]. Neuroinflammation has thus become considered as a common prominent feature among a variety of neurodegenerative disorders. Attention on the pathogenetic role of neuroinflammation has, over the past two decades, been expanded to psychiatric disorders. Immunohistochemistry and positron emission tomography studies have revealed microglial activation in the brain of patients with schizophrenia [11,12,13] and major depression (MD) [14,15], the representative endogenous psychoses. Neuroinflammation could thus be important in many pathological conditions of the brain, including both neurodegenerative disorders and psychiatric disorders (hereinafter referred to as neuropsychiatric disorders in this article). 

Based on the aforementioned findings, chronic inflammation can be regarded as a common denominator of periodontitis and neuropsychiatric disorders. Specifically, neuroinflammation may causally link periodontitis to the clinical onset and development of neuropsychiatric disorders. Furthermore, through the biological mechanism of chronic inflammation, periodontitis could causally affect neuropsychiatric disorders, especially MD, because of its psychosocial effects, such as shame, loneliness, impaired quality of life (QOL), and impaired social status [16]. Nonetheless, this review article focuses on the biological and epidemiological evidence for possible causal links of periodontitis to the selected neuropsychiatric disorders, namely AD, MD, PD, and schizophrenia. This article also discusses an association between periodontitis and the neurological event of ischemic stroke.

## 2. How Does Periodontitis Cause Neuroinflammation? 

**Figure 1:**
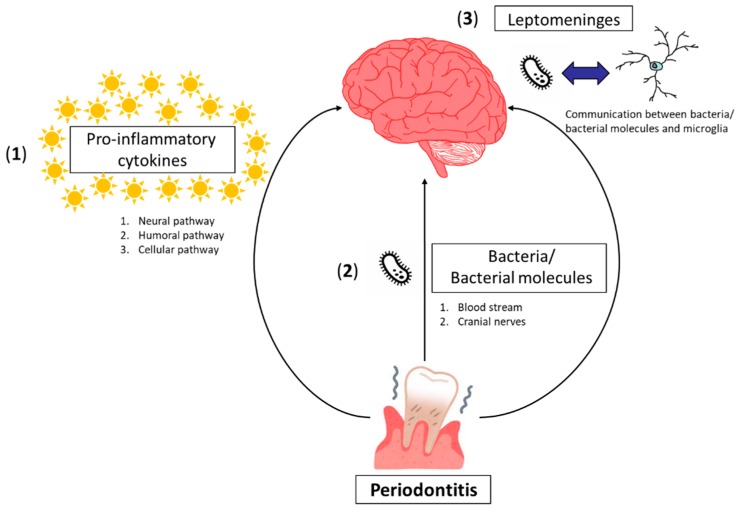
Scheme for presumed mechanisms by which periodontitis causes neuroinflammation. These consist of three possibilities as follows: (**1**) Peripheral pro-inflammatory cytokines associated with periodontitis communicate with the brain via the neural pathway, humoral pathway, and cellular pathway. (**2**) Periodontal bacteria/bacterial molecules can directly invade the brain either through the blood stream or via cranial nerves. (**3**) Communication between periodontal bacteria/bacterial molecules and brain-resident microglia could occur through the leptomeninges.

Neuroinflammation is a key pathogenetic connector between periodontitis and neuropsychiatric disorders. The biological mechanisms by which periodontitis causes neuroinflammation can be presumed to consist of three possibilities, as follows (Figure 1).

(**1**) Neuroinflammation can be caused by peripheral pro-inflammatory cytokines generated in systemic inflammation induced by periodontitis without the contact of periodontal bacteria/bacterial molecules with the brain tissue via three pathways, i.e., the neural pathway, the humoral pathway, and the cellular pathway. Through the neural pathway, systemic cytokines directly activate primary afferent nerves, such as the vagus nerve. The signal reaches the primary and secondary projection of the neural pathway, reaching first the nucleus tractus solitaries and subsequently, various hypothalamic brain nuclei [17]. It has been shown that subdiaphragmatic vagotomy blocks the LPS-induced sickness behavior in rats [18], while it does not affect the LPS-induced synthesis of pro-inflammatory cytokines at the periphery. The humoral pathway involves the choroid plexus and circumventricular organs, which lack an intact blood–brain barrier (BBB). These leaky regions may be access points for circulating pro-inflammatory cytokines to enter into the cerebral parenchyma by volume diffusion and elicit downstream signaling events, which are important in altering brain function [19]. The cellular pathway implicates systemic inflammation in association with both activation of endothelial cells (CECs) and an increase in circulating monocytes [19]. Systemic pro-inflammatory cytokines activate CECs, expressing receptors for TNF-α and IL-1β, which in turn, signal to the perivascular macrophages located immediately adjacent to CECs [20]. These perivascular macrophages subsequently communicate with microglia and thus lead to microglial activation. Activated microglia secrete not only pro-inflammatory cytokines but also proteases and chemokines, including monocyte chemoattractant protein (MCP)-1. MCP-1 is supposed to be responsible for the recruitment of monocytes into the motor cortex, hippocampus, and basal ganglia regions, areas of the brain known to be involved in the control of behavior [21].

(**2**) Periodontal bacteria/bacterial molecules can directly invade the brain either through the blood stream or via cranial nerves. In periodontitis, a periodontal pocket is filled with periodontal bacteria/bacterial molecules that form biofilms. Since periodontal bacteria are capable of invading an intact pocket epithelium, periodontal bacteria/bacterial molecules can gain access to the circulation [22]. It has been shown that LPS deteriorates the BBB and increases its permeability through abnormal activation of matrix metalloproteinase [23]. Circulating periodontal bacteria/bacterial molecules could then penetrate into the brain through the compromised BBB. In fact, *P. gingivalis* DNA has been identified by quantitative polymerase chain reaction (qPCR) in the brain of mice orally infected with *P. gingivalis* [24], and *P. gingivalis*-derived LPS has been detected in the brains of AD patients [25]. Circulating periodontal bacteria/bacterial molecules can also enter the brain through the circumventricular organs and choroid plexuses that lack the contiguous BBB. The cranial nerve may be another entry route for periodontal pathogens into the brain. The olfactory and trigeminal nerves are known to be used by periodontal bacteria to bypass the BBB [26]. The detection of oral *Treponemas* in the trigeminal ganglia supports the idea of neural pathways [27]. Via any of these pathways, infiltration of periodontal bacteria/bacterial molecules into the brain could result in inflammatory activation of microglia, since it has been demonstrated that either *P. gingivalis* infection or LPS of *P. gingivalis* activates microglia in vitro to produce pro-inflammatory cytokines, such as tumor necrosis factor (TNF)-α, interleukin (IL)-1β and IL-6 [28,29].

(**3**) The leptomeninges could be a site of communication between periodontal bacteria and brain-resident microglia. The leptomeninges covers the brain parenchyma surface and provides a physical boundary at the cerebrospinal fluid (CSF)-blood barrier. Leptomeningeal cells express Toll-like receptors (TLRs) 2 and 4 that are the receptors for *P. gingivalis* LPS. Leptomeningeal cells can be activated by circulating *P. gingivalis* LPS and subsequently produce pro-inflammatory cytokines for the brain [30,31]. The pro-inflammatory cytokines released from leptomeningeal cells activate microglia to evoke neuroinflammation. Accordingly, the leptomeninges could be harmful by transducing peripheral inflammation, including periodontitis, into neuroinflammation.

## 3. Alzheimer’s Disease

AD is currently the best-known neuropsychiatric disease that is associated with periodontitis based on clinical and experimental evidence that is accumulating rapidly. Recent epidemiological studies have pointed out that periodontitis significantly elevates the risk for AD. A prospective pilot study using qPCR identified *P. gingivalis* DNA in the CSF in seven of the 10 clinically diagnosed AD patients who had mild to moderate cognitive impairment [24]. A cross-sectional study reported that plasma TNF-α and antibodies against periodontal bacteria were elevated in AD patients relative to normal controls and were independently associated with AD [32]. Another cross-sectional study demonstrated that the increased serum levels of TNF-α and IL-6 in patients with AD were significantly associated with periodontitis [33]. A case-control study established a significant association between AD and the increased number of deep periodontal pockets [34]. A retrospective matched cohort study on 9291 patients with periodontitis showed that chronic periodontitis exposure for 10 years was associated with a 1.707-fold increase in the risk of developing AD [35]. Another historical cohort study with the larger sample size of 262,349 participants who suffered from chronic periodontitis supports this finding [36]. Prospective cohort studies with relatively small sample sizes demonstrated that serum IgG antibody levels to periodontitis bacteria, such as *P. gingivalis*, *Tannerella forsythia* and *Treponema denticola* (the so-called “red complex”), were significantly increased in baseline serum drawn from subjects who were diagnosed with AD in later years compared to controls [37,38]. Even after the onset of AD, periodontitis may exacerbate cognitive impairment. A six-month observational cohort study tested cognitive function and serum pro-inflammatory markers in 52 patients with mild to moderate AD. The study showed that the presence of periodontitis at baseline was associated with a six-fold increase in the rate of cognitive decline in participants over the six-month follow-up period, and was also associated with a relative increase in the pro-inflammatory state over that period [39]. A meta-analysis based on one cross-sectional study [40] and two case-control studies [41,42], which have not been previously mentioned, and assessed as at a low risk of bias, came to the conclusion that periodontitis is significantly associated with AD (OR 1.69, 95% CI 1.21–2.35) [43]. Furthermore, severe forms of periodontitis show a more intense association with AD (OR 2.98, 95% CI 1.58–5.62) [43]. An interesting study comparing periodontal conditions between countries reported that periodontitis was more prevalent in Germany and that elderly German subjects had significantly more severe periodontal conditions and fewer remaining teeth compared to those in Japan, even after adjustment of the comprehensive risk factor [44]. Accordingly, it would be tempting to examine whether the AD prevalence in Germany is significantly higher than that of Japan by a direct comparison with the adjustment of confounding factors.

Growing evidence based on animal studies also strengthens a possible causal link of periodontitis to AD. Chronic intraperitoneal injection of *P. gingivalis* LPS (1 mg/kg/day, daily for 5 weeks) has been demonstrated to cause learning and memory deficits accompanied with intracellular amyloid β (Aβ) accumulation in neurons and microglial activation (i.e., increased expression of IL-1β and TLR2 restricted to microglia) in the hippocampus in middle-aged mice (12 months old), but not in young mice (2 months old) or in middle-aged cathepsin-B knockout mice [29]. Therefore, cathepsin B, known as an amyloid precursor protein (APP) secretase, may play a critical role in the periodontitis-exacerbated AD and could be a therapeutic target. Even a single intraperitoneal injection of *P. gingivalis* LPS (5 mg/kg) into 8-week old mice has been shown to impair spatial learning and memory with neuroinflammation (i.e., microglial activation, astrocytic activation, and increased expression of TNF-α, IL-1β, and IL-6 in the cortex and/or hippocampus) and activation of the TLR4/nuclear factor-kappa B (NF-κB) signaling pathway (i.e., up-regulation of TLR4, CD14, IL-1 receptor-associated kinase 1, and phospho-p65 in the cortex) [45]. These behavioral and immuno-biochemical findings were considerably abolished by the TLR4 inhibitor TAK242, suggesting that the *P. gingivalis* LPS-induced cognitive dysfunction and neuroinflammation are mediated by the TLR4/NF-κB signaling pathway [45]. Interestingly, this study also tested *E. coli* LPS and reported that either *P. gingivalis* LPS or *E. coli* LPS caused both cognitive impairment and neuroinflammation and there was no significant difference between the effects of the two LPS species [45]. Experimental chronic periodontitis, caused when live *P. gingivalis* (ATCC33277) was given by oral gavage every 48 h over 6 weeks, has been shown to impair learning and memory and elicit neuroinflammation (i.e., increased expression of TNF-α, IL-1β and IL-6 in the brain) in middle-aged mice (12 months old), although not in young individuals (4 weeks old) [46]. Experimental chronic periodontitis, induced by repeated oral application of another live *P. gingivalis* W83 every 48 h over 22 weeks, has been demonstrated to increase extracellular Aβ42 amyloid plaques, ser396 residue of tau protein phosphorylation, neurofibrillary tangle formation, and neuroinflammation (i.e., microglial activation, astrocytic activation, and increased expression of TNF-α, IL-1β and IL-6) in the hippocampus of 6-week old mice [47]. Currently, there is only one study that employed APP-transgenic (Tg) mice and the study implies that periodontitis exacerbates the hallmark pathology and symptoms of AD [48]. Specifically, APP-Tg mice with periodontitis induced by oral infection with *P. gingivalis* ATCC33277, showed greater deposition of Aβ40 and Aβ42 amyloid plaques in both the hippocampus and cortex and increased brain expression of TNF-α and IL-1β, compared with sham-infected APP-Tg mice. Furthermore, cognitive function was significantly impaired in the periodontitis-induced APP-Tg mice relative to sham-infected APP-Tg mice [48]. 

Postmortem studies have indicated the potentially causal presence of periodontopathic virulence products in AD brains. LPS from *P. gingivalis* was detected in the brain of four out of 10 AD cases by immunofluorescence and Western blot (WB) analyses, whereas *P. gingivalis* LPS was not detected in 10 age-matched non-AD controls. Dominiy et al. (2019) have performed a seminal study, identifying *P. gingivalis* DNA and gingipains, toxic proteases secreted from *P. gingivalis* in AD brains. Immunohistochemical analyses using tissue microarrays showed that gingipain immunoreactivity in AD brains was significantly greater than that in sex- and age-matched non-AD brains, and that gingipain immunoreactivity significantly correlates with tau and ubiquitin loads and AD diagnosis [24]. Using qPCR, the authors identified *P. gingivalis* DNA in the AD brains which were lysine gingipain-positive in WB and immunoprecipitated analyses [24]. In addition to postmortem brain studies, they carried out in vivo studies using wild-type mice and gingipain-knockout mice that were orally infected with *P. gingivalis* W83 every other day for 42 days. Colonization of *P. gingivalis* and Aβ42 levels were increased in the brains of the infected wild-type mice, while the colonization and Aβ42 levels were decreased in the brains of either the infected wild-type mice treated with the gingipain inhibitor COR119 or of the gingipain-knockout mice [24]. Because these findings suggest that gingipain inhibition reduces the *P. gingivali* load and Aβ42 production in the brain, gingipain inhibitors could have therapeutic potential for patients with both AD and periodontitis. 

## 4. Major Depression

MD has also attracted attention and is currently the second elucidated neuropsychiatric disease with respect to its reciprocal association with periodontitis. A recent cross-sectional study on 108 subjects reported that patients with periodontitis showed a significantly higher comorbidity rate of depression (62.5%) compared to periodontally healthy subjects (38.86%), excluding smokers, pregnant women, subjects with systemic pathologies, and subjects taking antidepressants [49]. MD is susceptible to psychosocial factors and periodontitis could increase the risk for developing MD through the psychosocial effects that stem from periodontitis-causing oral troubles, such as halitosis, poor oral hygiene, and edentulousness [16]. Patients with malodorous wounds and poor oral hygiene often experience social isolation, depression, shame, and poor appetite, all of which have a negative impact on their QOL [50]. Also, tooth loss negatively affects the patients’ QOL since it worsens not only chewing function, but also body image, self-esteem, and social status [16,51]. Because of all this, many early studies in this field were performed from a psychosocial viewpoint [52,53]. 

Although several studies have recently focused on the biological relationship between periodontitis and MD, most of them investigated periodontitis as an outcome influenced by MD [54,55]. The effects of antidepressants, which are known to possess anti-inflammatory and immunomodulatory properties [56,57], on chronic periodontitis have been studied in both MD animal models (systematically reviewed in [58]) and in MD patients [59,60]. The majority has established the therapeutic effects of antidepressants on chronic periodontitis. A recent cross-sectional study made by Gomes et al. (2018), who hypothesized that increased root canal LPS accompanying chronic apical periodontitis causes MD, showed that patients with periodontitis and MD had highly increased root canal endotoxin levels relative to patients with periodontitis without MD or normal controls. This study demonstrated a strong positive association between periodontitis or root canal endotoxin levels and the severity of MD, suggesting that the association between periodontitis and MD is attributable, at least in part, to root canal endotoxin levels [61]. In the study, periodontitis-related MD was accompanied with elevated levels of oxidative and nitrosative stress index, including nitric oxide metabolites and hydroperoxides, which are supposed to play a role in the pathogenesis of MD [62]. A recent cohort study composed of a high methodological quality with more than 60,000 participants and a 10-year follow-up period also supports the feasible causal link of periodontitis to MD. The study showed a higher incidence of subsequent depression in the periodontitis group (*n* = 12,708) than in the non-periodontitis group (*n* = 50,832) (HR 1.73, 95% CI 1.58–1.89), after adjustment of sex, age and comorbidities [63]. This result suggests that periodontitis is an independent risk factor for subsequent MD regardless of sex, age, and the comorbidities except for diabetes, alcohol abuse, and cancer. On the other hand, in a meta-analysis on four cross-sectional studies [64,65,66,67] that were assessed as moderate-high quality of the evidence and considered periodontitis as the outcome and MD as the exposure, the pooled estimate does not show association between periodontitis and MD (OR 0.96, 95% CI 0.84–1.10) [68]. Therefore, more prospective cohort studies that test periodontitis as the independent variable and MD as the outcome, or interventional studies, such as studies that determine the effects of periodontal treatment on MD, are clearly warranted to elucidate the causal relationship between periodontitis and MD. 

## 5. Parkinson’s Disease

Compared with AD and MD, any correlation between PD and periodontitis has, traditionally, been less effectively understood. Nevertheless, a few studies have noted an increased prevalence of periodontal disease among individuals with PD relative to age-matched controls [69,70]. PD causes motor disturbance (due to tremor, rigidity, akinesia, and involuntary movement), apathy, and cognitive impairment, all of which appear to make it difficult for patients to maintain daily oral hygiene. Therefore, periodontitis can be considered as a consequence of the poor oral hygiene related to clinical symptoms of PD. Recent epidemiological studies have investigated whether periodontitis increases the risk for developing PD. Chen et al. (2017) conducted a population-based retrospective matched-cohort study and reported that individuals with newly diagnosed periodontitis (*n* = 5396) had an increased risk of subsequent PD compared to individuals without periodontitis (*n* = 10,792), regardless of sex, age, comorbidities, and urbanization levels (HR 1.431, 95% CI 1.141–1.794) [71]. The authors also examined the effect of periodontal treatment on developing PD. Their population-based nested case-control study demonstrated that among individuals without periodontitis aged 40–69 (*n* = 5552), dental scaling over five consecutive years showed a protective effect against PD development, relative to individuals who did not undergo dental scaling (OR 0.204, 95% CI 0.047–0.886) [72]. Moreover, among individuals with periodontitis aged ≥70 (*n* = 3377), the discontinued scaling (i.e., not five consecutive years) or no treatment were significant risk factors for developing PD [35]. These findings suggest that early and consecutive dental scaling could prevent the development of PD. Although these seminal epidemiological studies imply a feasible causal link of periodontitis to PD and a preventive effect of periodontal treatment on PD development, experimental studies for verifying these concepts are lacking at the present time. Therefore, additional epidemiological studies and experimental studies along this line are required. 

## 6. Schizophrenia

Evidence for a significant relationship between periodontitis and schizophrenia has not yet been accumulated. Only one cross-sectional study with a small sample size has concluded that patients with schizophrenia have a high risk of periodontitis and there is an even higher risk in those who are taking antipsychotics that reduce salivary secretion and cause xerostomia [73]. Intriguingly, human genome/gene analysis on insertion/deletion (D) polymorphism in the angiotensin-converting enzyme (ACE) gene has indicated that the D allele is a protective factor against schizophrenia [74] and chronic periodontitis [75]. Accordingly, the ACE D allele may be a clue to reveal any biological and reciprocal connection between these two diseases. 

## 7. Ischemic Stroke 

Epidemiological studies also suggest an association between periodontitis and ischemic stroke. Stroke is the second most common cause of mortality worldwide and approximately 80% of strokes are caused by focal cerebral ischemia [76]. A recent meta-analysis [76] has shown a significant association between periodontitis and ischemic stroke based on three cohort studies [77,78,79] (pooled RR 2.52, 95% CI 1.77–3.58) and five case-control studies (pooled RR 3.04, 95% CI 1.10–8.43) [80,81,82,83,84]. Chi et al. (2019) examined mice with both experimental periodontitis induced by periodontal injection of LPS and photothrombotic ischemia. The study has demonstrated that chronic periodontitis exacerbates ischemic stroke through increasing the activation of microglia/astrocytes and the expression of nod-like receptor protein 3 inflammasome and IL-1β [85], suggesting that chronic periodontitis is a driving force for neuroinflammation associated with ischemia.

## 8. Conclusions

The relationship between periodontitis and neuropsychiatric disorders, in particular AD, has recently attracted researchers’ attention, and the evidence for their considerable reciprocal association has been accumulated. Specifically, various clinical and experimental studies imply the potentially causal link of periodontitis to neuropsychiatric disorders, and neuroinflammation seems to be a key pathological connector between them. After establishment of such a link, a broad spectrum of neuropsychiatric disorders associated with neuroinflammation may be preventable and modifiable by simple daily dealings for oral hygiene, even though the notion that periodontitis is a risk factor for neuropsychiatric disorders remains to be effectively substantiated.
